# Short-Term Effect of One-Anastomosis Gastric Bypass on Essential Fatty Acids in the Serum of Obese Patients

**DOI:** 10.3390/nu12010187

**Published:** 2020-01-09

**Authors:** Adriana Mika, Maciej Wilczynski, Alicja Pakiet, Lukasz Kaska, Monika Proczko-Stepaniak, Marta Stankiewicz, Piotr Stepnowski, Tomasz Sledzinski

**Affiliations:** 1 Department of Pharmaceutical Biochemistry, Faculty of Pharmacy, Medical University of Gdansk, 1 Debinki, 80-211 Gdansk, Poland; tsledz@gumed.edu.pl; 2 Department of Environmental Analytics, Faculty of Chemistry, University of Gdansk, Wita Stwosza 63, 80-308 Gdansk, Poland; alicja.pakiet@phdstud.ug.edu.pl (A.P.); piotr.stepnowski@ug.edu.pl (P.S.); 3 Department of General, Endocrine, and Transplant Surgery, Faculty of Medicine, Medical University of Gdansk, Smoluchowskiego 17, 80-214 Gdansk, Poland; wilczynskimj@gmail.com (M.W.); lukasz.kaska@wp.pl (L.K.); mproczko@gumed.edu.pl (M.P.-S.); 4 Department of Clinical Nutrition, Faculty of Health Sciences, Medical University of Gdansk, Debinki 7, 80-211 Gdansk, Poland; marta.stankiewicz@gumed.edu.pl

**Keywords:** essential fatty acids, odd-chain fatty acids, obesity, one-anastomosis gastric bypass

## Abstract

One-anastomosis gastric bypass is a promising type of bariatric surgery, but it may lead to a deficiency in important nutrients, such as fatty acids. The short-term effects of one-anastomosis gastric bypass on serum fatty acids have not been studied thus far. Therefore, the aim of this study was to determine the effect of one-anastomosis gastric bypass on serum fatty acid composition two weeks after surgery. This study included 38 patients who underwent one-anastomosis gastric bypass as surgical treatment for morbid obesity. Serum fatty acid composition was analyzed before and two weeks after surgery using gas chromatography–mass spectrometry. We observed a decrease in essential polyunsaturated fatty acids (*p* < 0.001 for linolenic acid and *p* < 0.001 for linoleic acid) and odd-chain fatty acids (*p* = 0.004) in the serum of obese patients shortly after a one-anastomosis gastric bypass. Considering the benefits of the aforementioned fatty acids for human health, the implementation of a fatty-acid-rich diet or the use of supplementation may be recommended for patients immediately after one-anastomosis gastric bypass.

## 1. Introduction

Bariatric surgery (BS) is currently the most effective method for obesity treatment [1]. However, some studies demonstrated negative BS effects, such as limited nutrient uptake, hypoglycemia, and osteoporosis [1]. Others focused on the advantages of BS, including diabetes remission and a reduction in the risk of coronary disease and cancer [2]. Some emphasized that the effect of BS on obese patients depends on the surgical technique used [3]. The status of BS as a weight-reduction treatment is evolving to reflect its potential use as a treatment for metabolic disorders. It is worth focusing on patient comfort and health in the postsurgical period, when the body is weak and requires nutritional support. Fatty acids (FAs) are important molecules that act as substrates for cellular-energy production, cell-membrane formation, and cell signaling [4]. Some FAs, despite existing in small quantities, play an important role in the human body. Examples of such FAs are omega-3 polyunsaturated FAs (n-3 PUFAs), which have anti-inflammatory properties, branched-chain saturated FAs (BCFAs), which were shown to display antitumor effects, and odd-chain saturated FAs (OCFAs), which were shown to exert antibiotic, anti-inflammatory, and antioxidant effects [4,5]. Reductions in the levels of some FAs in the serum of obese patients were observed after BS due to decreased postoperative dietary intake [6]. Interestingly, reports to date described the short- and long-term effects of laparoscopic sleeve gastrectomy (LSG), biliopancreatic diversion with a duodenal switch (BPDDS) [6], and Roux-en-Y gastric bypass (RYGB) on serum FA composition [7,8]. However, the short-term period after one-anastomosis gastric bypass (OAGB) has not been investigated. OAGB is a promising type of BS that improves metabolic parameters [9]. Therefore, the aim of this study was to determine the effect of OAGB on the levels of particular FAs in the serum of obese patients two weeks after BS. We also compared serum FA composition in obese patients before and after surgery with lean control subjects.

## 2. Materials and Methods

### 2.1. Study Subjects

This study included 38 patients (32 females and 6 males) who underwent OAGB as a surgical treatment for morbid obesity. Thirteen patients had type 2 diabetes mellitus. Blood was collected in the morning on the day of the surgery. Obese patients were on a low-calorie diet (high-protein, low-fat, and low-carbohydrate meals) for 2–3 months prior to surgery. Patients did not take any weight-loss medications before surgery. In every case, a 180 cm segment of the small intestine was removed, and a standardized 50 mL stomach pouch was created. All of the enrolled study patients underwent a follow-up visit 2 weeks after surgery. The control group consisted of 30 lean individuals (15 males and 15 females) without metabolic disorders. Fasting blood samples from all study subjects were collected in the morning to assay serum FA composition. During the first 2 weeks after surgery, as the stomachs began to heal, it was recommended that patients consume low-sugar, low-fat, and high-protein liquids and mashed foods, i.e., cream soup consisting of poultry, eggs, and fish. Patients were required to restrict their caloric intake, often to less than 500 kcal per day. Patients were advised to consume natural (unsweetened) milk products such as yogurt, buttermilk, kefir, and vegan milk (i.e., sesame and soya). Pure protein powder was added to these products. It was instructed that food should be frequently consumed and in small portions, i.e., every 3 h. Water, noncarbonated flavored water, ice cubes, tea without sugar, juices diluted with water, sugarfree jelly, protein-containing drinks, and protein-containing fruit drinks (e.g., smoothies) were also recommended. The most important recommendation was that protein intake should be greater than 60 g/day. Vitamin regimens specific for bariatric patients were used from the first postoperative day. Omega acid supplementation was not routinely recommended. The study was performed in agreement with the principles of the Declaration of Helsinki of the World Medical Association. The study protocol was approved by the Local Bioethics Committee at the Medical University of Gdansk (decision no. NKBBN/493/2016), and written informed consent was obtained from all participants.

Characteristics of lean subjects and obese patients before and 2 weeks after OAGB are presented in Table 1.

### 2.2. Serum Fatty Acid Composition Analysis by Gas Chromatography–Mass Spectrometry

Lipids were extracted from serum aliquots using a chloroform–methanol mixture (2:1, *v/v*) and dried under a nitrogen stream. Samples were hydrolyzed with 0.5 M KOH in methanol at 90 °C for 3 h and acidified with 0.5 mL of 6 M HCl. Following this, 1 mL of water was added, and FAs were extracted using 3 × 1 mL n-hexane. FAs were then derivatized into FA methyl esters and analyzed using gas chromatography–electron ionization–mass spectrometry as previously described [9]. FA identification was carried out using reference standards (37 FAME MIX, Sigma Aldrich, Saint Louis, MO, USA) and the National Institute of Standards and Technology 2011 reference library. The used internal standard was 19-methylarachidic acid.

### 2.3. Statistics

Differences between variables measured before and after surgery in obese subjects were analyzed using paired *t*-tests. Differences between obese subjects and lean controls were analyzed using 2-tailed *t*-tests. Data are presented as mean ± standard deviation (SD). Calculations were performed using Microsoft Excel (Microsoft, Redmond, WA, USA).

## 3. Results

We observed significant decreases in body mass index and fasting serum glucose concentration after OAGB. Concentrations of albumin and total protein increased (Table 1). The increase in total protein was most likely a result of the high-protein diet recommended after surgery. The implementation of a high-protein diet following surgery, prior to the significant reduction in body and muscle mass, may have resulted in the observed increase in serum protein concentrations.

Obese subjects demonstrated severely disturbed serum FA composition compared to the control subjects. This included a decrease in BCFAs and an increase in monounsaturated FAs (MUFAs) (Table 2). Obese patients also showed a decrease in some polyunsaturated FAs (PUFAs), including linolenic acid (18:3 n-3), eicosatetraenoic acid (EPA; 20:5 n-3), and eicosatetraenoic acid (20:4 n-3) (Table 2); these FAs are omega-3 PUFAs. In turn, obese patients showed a decrease in linoleic acid (18:2 n-6) and eicosadienoic acid (20:2 n-6), which are omega-6 PUFAs (Table 2).

Serum OCFA levels were significantly decreased compared to the controls in the two weeks following OAGB (Table 2). Significant decreases were also observed in 18:3 n-3 and 18:2 n-6. These are essential FAs that cannot be synthesized in the human body and must be obtained from the diet. We also noted greater decreases in EPA, 20:4 n-3, and 20:2 n-6 two weeks after OAGB (Table 2). BCFA levels did not significantly change in the period after OAGB, but they remained significantly lower compared to controls. By contrast, we observed increases in arachidonic acid (20:4 n-6) two weeks after OAGB. This change is associated with inflammation. We also noted changes in some saturated FAs and MUFAs, but total saturated FAs and MUFAs did not significantly change (Table 2).

## 4. Discussion

Our study revealed that OAGB leads to a short-term decrease in some FAs (two weeks). These decreases were seen in diet-derived FAs—OCFAs and essential PUFAs. This suggests that a deficiency in these FAs is most likely the effect of decreased dietary intake after BS. Lin et al. [6] arrived at the same conclusion in their study of short-term BPDDS and LSG effects. The aforementioned types of FAs play an important functional role in the human body, and their depletion can be unfavorable for patients shortly after surgery. OCFAs mainly originate from the milk and meat of ruminants. They display anti-inflammatory and antioxidant properties and reduce cardiovascular-disease risk [10]. Given these properties, OCFA deficiency in patients after BS should be avoided. This is the first study to show the short-term effects of BS on serum OCFAs in obese subjects. Our recent study of the long-term effects of BS did not show significant differences in serum OCFA levels before and six to nine months after BS [9]. This suggests that their levels eventually normalized after BS.

PUFAs n-3 and n-6 play many important functional roles in the human body, including cardioprotection and the regulation of inflammation [4]. Our previous study demonstrated that obese subjects showed decreased serum levels of n-3 and n-6 PUFAs [5]. PUFAs 18:2 n-6 and 18:3 n-3 cannot be synthesized in humans, but they can be converted into other PUFAs by elongases and desaturases [4]. Hence, deficiencies in these two FAs can have particularly severe consequences. Lin et al. also observed a reduction in the serum levels of 18:3 n-3, 18:2 n-6, and EPAs in obese patients as early as three days after BPDDS and LSG. Forbes et al. [8] observed a decrease in the aforementioned PUFAs one month after RYGB, but Walle et al. [7] observed slight increases in n-3 and n-6 PUFAs one year after RYGB. Our previous results showed modest reductions in n-3 and n-6 PUFAs six to nine months after OAGB. These results suggest that PUFA deficiency may be resolved a long time after BS. 

Despite the finding that BCFA levels did not change in the two weeks following OAGB, they remained lower than those in lean subjects. Previous studies also suggested the beneficial effects of this type of FA. These include antitumor effects and inverse correlations with inflammation, dyslipidemia, and insulin resistance in obese subjects [5]. Thus, BCFA deficiency is another unfavorable condition that persists in patients after OAGB. Serum BCFA levels return to those seen in lean subjects six to nine months after OAGB [9]. The short-term effects of BS on BCFA levels have also not been studied thus far.

The limitations of our study include the lack of other time points of serum FA composition analysis after BS, and the relatively small cohort used. However, despite this sample size, the statistical significance of the results was quite robust and therefore convincing.

## 5. Conclusions

Our results showed short-term reduction in the serum concentrations of OCFAs and essential PUFAs after OAGB. BCFA levels also remained low after OAGB. Considering the benefits of the aforementioned FAs in human health, the implementation of a diet rich in these FAs or the use of supplementation is recommended for obese patients immediately after a bariatric procedure.

## Figures and Tables

**Table 1:** Characteristics of lean controls and obese patients before and 2 weeks after one-anastomosis gastric bypass (OAGB).

	Lean Controls (LC)	Pre-OAGB	Two Weeks Post-OAGB	*p* (Pre-OAGB vs. LC)	*p* (PreOAGB vs. Two Weeks Post-OAGB)	*p* (Two Weeks Post-OAGB vs. LC)
Age (years)	49.97 ± 10.92	48.09 ± 9.57	-	0.461	-	-
BMI (kg/m^2^)	24.9 ± 2.68	37.1 ± 2.73	33.8 ± 2.33	<0.001	<0.001	<0.001
Total cholesterol (mg/dL)	218 ± 44.0	206 ± 34.4	212 ± 37.5	0.251	0.487	0.566
Triglycerides (mg/dL)	100 ± 53.4	114 ± 38.5	120 ± 32.7	0.231	0.406	0.086
Glucose (mg/dL)	94.6 ± 24.6	132 ± 35.8	111 ± 20.7	<0.001	0.001	0.004
Albumin (g/L)	45.7 ± 2.99	42.8 ± 3.60	49.9 ± 4.45	<0.001	<0.001	<0.001
Total protein (g/L)	72.6 ± 5.55	69.6 ± 6.11	78.4 ± 7.48	0.043	<0.001	<0.001

**Table 2:** Serum fatty acid composition (%) in lean controls and obese subjects before and two weeks after bariatric surgery.

	Lean Control (LC)	Pre-OAGB	2 Weeks Post-OAGB	*p* (Pre-OAGB vs. LC)	*p* (Pre vs. Post-OAGB)	*p* (Post-OAGB vs. LC)
	Mean % of Total Fatty Acid (FA) ± SD					
16:0	23.21 ± 1.77	24.55 ± 1.75	26.06 ± 1.46	0.001	<0.001	<0.001
18:0	7.30 ± 0.72	6.19 ± 0.60	5.52 ± 0.80	<0.001	<0.001	<0.001
Other ECFA	1.82 ± 0.36	1.59 ± 0.39	1.26 ± 0.32	0.011	<0.001	<0.001
**ECFA**	**32.33 ± 1.87**	**32.33 ± 1.89**	**32.84 ± 1.73**	**0.995**	**0.136**	**0.223**
15:0	0.23 ± 0.05	0.26 ± 0.06	0.24 ± 0.06	0.073	0.334	0.417
17:0	0.24 ± 0.04	0.24 ± 0.04	0.22 ± 0.04	0.226	<0.001	<0.001
Other OCFA	0.15 ± 0.04	0.10 ± 0.03	0.08 ± 0.03	<0.001	<0.001	<0.001
**OCFA**	**0.64 ± 0.10**	**0.60 ± 0.10**	**0.55 ± 0.11**	**0.146**	**0.004**	**<0.001**
**Anteiso-BCFA**	0.20 ± 0.05	0.13 ± 0.04	0.13 ± 0.06	<0.001	0.967	<0.001
**Iso-BCFA**	0.20 ± 0.06	0.014 ± 0.04	0.13 ± 0.05	<0.001	0.231	<0.001
**Total BCFA**	**0.42 ± 0.10**	**0.29 ± 0.08**	**0.28 ± 0.11**	**<0.001**	**0.673**	**<0.001**
**Total SFA**	**33.39 ± 1.84**	**33.22 ± 1.93**	**33.66 ± 1.77**	**0.710**	**0.211**	**0.505**
16:1	3.02 ± 0.97	3.50 ± 1.00	3.43 ± 1.14	0.035	0.623	0.092
18:1	26.61 ± 3.09	29.23 ± 3.14	29.94 ± 2.47	<0.001	0.184	<0.001
Other MUFA	0.54 ± 0.13	0.53 ±0.11	0.61 ± 0.11	0.968	<0.001	<0.001
**MUFA**	**30.16 ± 3.53**	**33.31 ± 3.59**	**34.03 ± 2.93**	**<0.001**	**0.257**	**<0.001**
18:3 n-3	0.34 ± 0.11	0.24 ± 0.11	0.15 ± 0.10	<0.001	<0.001	<0.001
20:5 n-3 (EPA)	1.09 ± 0.72	0.76 ± 0.45	0.54 ± 0.18	0.018	0.001	<0.001
20:4 n-3	0.10 ± 0.03	0.06 ± 0.02	0.04 ± 0.02	<0.001	<0.001	<0.001
22:6 n-3 (DHA)	1.14 ± 0.44	1.36 ± 0.54	1.42 ± 0.39	0.053	0.445	0.004
Other n-3 PUFA	0.29 ± 0.05	0.34 ± 0.09	0.39 ± 0.09	0.003	<0.001	<0.001
**n-3 PUFA**	**2.97 ± 1.14**	**2.76 ± 0.95**	**2.54 ± 0.54**	**0.384**	**0.095**	**0.041**
18:2 n-6	26.24 ± 3.85	23.00 ± 3.21	20.63 ± 2.79	<0.001	<0.001	<0.001
20:4 n-6 (ARA)	5.61 ± 1.15	6.20 ± 2.00	7.85 ± 2.12	0.118	<0.001	<0.001
20:2 n-6	0.16 ± 0.03	0.11 ± 0.03	0.09 ± 0.03	<0.001	0.019	<0.001
Other n-6 PUFA	1.32 ± 0.25	1.31 ± 0.34	1.10 ± 0.26	0.944	<0.001	<0.001
**n-6 PUFA**	**33.32 ± 3.96**	**30.62 ± 1.93**	**29.68 ± 3.19**	**0.006**	**0.241**	**<0.001**

ECFA, even-chain saturated fatty acid; OCFA, odd-chain saturated fatty acid; BCFA, branched-chain saturated fatty acid; SFA, saturated fatty acid; MUFA, monounsaturated fatty acid; EPA, eicosapentaenoic acid; DHA, docosahexaenoic acid; DPA, docosapentaenoic acid; PUFA, polyunsaturated fatty acid; ARA, arachidonic acid; DGLA, dihomo-γ-linolenic acid; AdA, docosatetraenoic acid. Boldface—major groups of fatty acid.
